# Endoscopic Stent Placement Can Successfully Treat Gastric Leak Following Laparoscopic Sleeve Gastrectomy If and Only If an Esophagoduodenal Megastent Is Used

**DOI:** 10.1007/s11695-021-05467-x

**Published:** 2021-11-03

**Authors:** Franck Billmann, Aylin Pfeiffer, Peter Sauer, Adrian Billeter, Christian Rupp, Ronald Koschny, Felix Nickel, Moritz von Frankenberg, Beat Peter Müller-Stich, Anja Schaible

**Affiliations:** 1 Department of Surgery, University Hospital of Heidelberg, Im Neuenheimer Feld 420, D-69120 Heidelberg, Germany; 2 Interdisciplinary Endoscopic Center, University Hospital of Heidelberg, Im Neuenheimer Feld 420, D-69120 Heidelberg, Germany; 3 Krankenhaus Salem, Zeppelinstraße 11-33, D-69121 Heidelberg, Germany

**Keywords:** Bariatric surgery, Laparoscopic surgery, Gastrectomy, Postoperative complications, Stent, Treatment efficacy

## Abstract

**Purpose:**

Gastric staple line leakage (GL) is a serious complication of laparoscopic sleeve gastrectomy (LSG), with a specific mortality ranging from 0.2 to 3.7%. The current treatment of choice is stent insertion. However, it is unclear whether the type of stent which is inserted affects treatment outcome. Therefore, we aimed not only to determine the effectiveness of stent treatment for GL but also to specifically clarify whether treatment outcome was dependent on the type of stent (small- (SS) or megastent (MS)) which was used.

**Patients and Methods:**

A single-centre retrospective study of 23 consecutive patients was conducted to compare the outcomes of SS (n = 12) and MS (n = 11) for the treatment of GL following LSG. The primary outcome measure was the success rate of stenting, defined as complete healing of the GL without changing the treatment strategy. Treatment change or death were both coded as failure.

**Results:**

The success rate of MS was 91% (10/11) compared to only 50% (6/12) for SS (p = 0.006). An average of 2.3 ± 0.5 and 6.8 ± 3.7 endoscopies were required to achieve healing in the MS and SS groups respectively (p < 0.001). The average time to resumption of oral nutrition was shorter in the MS group (1.4 ± 1.1 days vs. 23.1 ± 33.1 days, p = 0.003).

**Conclusions:**

Stent therapy is only effective and safe for the treatment of GL after LSG if a MS is used. Treatment with a MS may not only increase treatment success rates but may also facilitate earlier resumption of oral nutrition and shorten the duration of hospitalization.

**Graphical Abstract:**

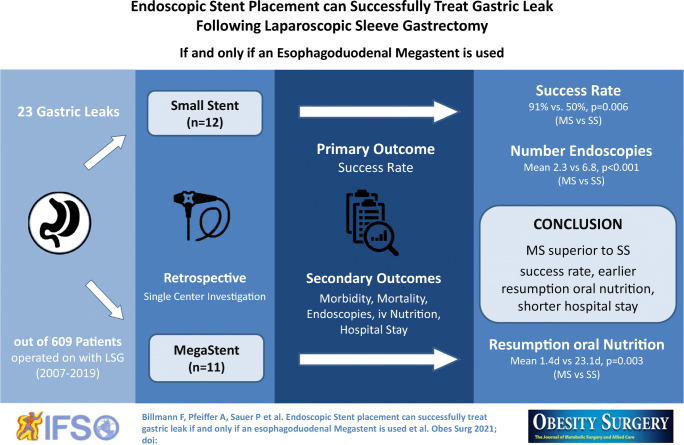

## Introduction

Laparoscopic sleeve gastrectomy (LSG) is now the most common bariatric procedure, accounting for almost 50% of all bariatric procedures performed worldwide [1]. However, the procedure is not without complications. In fact, gastric staple line leak (GL), also called staple line insufficiency, is a major complication of LSG, with an incidence of 2.1% (1.1–5.3%) [2]. This complication is not only serious but also potentially life-threatening with a specific mortality ranging from 0.2 to 3.7% [3]. GL is also associated with significant morbidity which results into additional medical costs, estimated to exceed 34,398 euros per patient for hospitalization and 41,284 euros per patient for out-patient treatment and follow-up [4].

The role of surgical revision is controversial [5–8]. Currently, the optimal treatment strategy for GL remains unclear but most surgeons favour endoscopic stent insertion, facilitating rapid and effective treatment [9–16] and delivering success rates ranging from 67 to 100% [9–22]. Several studies have clearly demonstrated the advantages of stent therapy which include [23] (i) a reduction in the number of procedures/interventions required, including endoscopies and stent changes, (ii) earlier resumption of oral nutrition and (iii) a significant reduction in the duration of hospital stay. The role of endoluminal vacuum therapy (E-Vac) therapy in the management of GL, particularly in the context of failed stent therapy [24], has recently received renewed attention.

A major obstacle to establishing the treatment of choice for post-LSG GL is the lack of carefully designed clinical studies. The studies performed to date have included heterogenous patient cohorts, examined the efficacy of stent placement for a variety of indications and lacked standardisation [25]. As a result, there is no firm evidence base which can be used determine which stent treatment is optimal [26, 27] in order to achieve the best clinical outcomes and to avoid the need for long-term E-Vac therapy.

Thus, we sought to determine the effectiveness of stent treatment for GL in a retrospective analysis of consecutively recruited patients and to clarify whether treatment outcome was dependent of which type of stent (small- (SS) or megastents (MS)) was inserted. We specifically investigated the success rates and morbidity following the insertion of the SS versus the MS. We conclude by comparing the efficacy of endoscopic stent insertion to other endoscopic treatment modalities, including E-Vac.

## Patients and Methods

### Study Design and Setting

This was a single-centre retrospective cohort study of 23 consecutively treated patients who underwent stent treatment for GL following LSG. The study was approved by the Institutional Review Board of the University of Heidelberg (ethic approval # S-044/2019) and performed in accordance with the Declaration of Helsinki and its later amendments. The study centre is a university surgical centre with extensive experience in bariatric and minimally invasive surgery (MIS), certified by the German Board of general and visceral surgery as reference centres for minimally invasive and bariatric surgery [28]. It has access to diagnostic, biochemical and imaging facilities necessary for the management of patients with morbid obesity. The operative interventions were performed as previously described [29] by the same group of experienced surgeons (n = 7). All participating surgeons had performed more than 50 MI sleeve gastrectomy procedures and had experience of at least 10 years in abdominal surgery. Patients were followed up on an in-patient basis for the duration of the primary treatment and subsequently in our out-patient clinic following discharge. The diagnosis of GL was made radiologically (computer tomography (CT) scanning with intravenous and oral contrast-enhancement) and confirmed endoscopically. The following stents used were (A) short stents (SS): Nicolai 25/20/25 mm, 85 mm fully covered; Nicolai 32/25/32 mm, 90 mm fully covered; Niti-S 36/28/36 mm, 100 mm fully covered; Niti-S 36/28/36 mm 120 mm fully covered (not covering the whole length of the gastric sleeve) and (B) megastents (MS) (specifically developed to cover the whole gastric sleeve from the gastroesophageal junction to the duodenum): Niti-S 32/24/32 mm, 230 mm fully covered; Niti-S 36/28/36 mm, 230 mm fully covered. The first MS was used in 2015. MS has completely replaced SS in our centre from 2016 onwards. This retrospective analysis was carried out for the period between 2007 and 2019 on the basis of a prospectively maintained bariatric database, specific to our centre in Heidelberg, the implementation and use of which was approved by our Institutional Review Board.

### Cohort and Follow-up

Patients included in our study were all diagnosed with morbid obesity (with a BMI > 35 kg/m^2^). We followed the DGAV-CAADIP pathway and procedural guidelines for the diagnosis and treatment of morbid obesity [30]. Follow-up for each patient began on the day of the operation. We only included patients who had undergone LSG and subsequently developed GL. All patients diagnosed with GL received treatment with intravenous antibiotics, initiated when GL was first diagnosed. All patients had undergone 6 months of conservative medical therapy and had received an endocrinological evaluation to rule out hormonal causes of obesity prior to surgery. In addition, patients underwent psychological evaluation to exclude severe eating disorders. Patients were excluded if (i) they did not complete a standard surgical follow-up of at least 1 year, (ii) the primary surgery was not performed in our centre and (iii) they were younger than 18 years of age, or if they were unable to give informed consent.

### Outcomes, Study Size and Bias

We gathered demographic, clinical and peri-operative data (including clinical, pathological, biochemical and comorbidity) for all of the patients included in this study.

The main outcome measure was the success rate of stent therapy. The following outcomes were also analysed: (1) the number of endoscopic procedures and the number of stent changes which were performed, (2) any complications linked to stent insertion, (3) the total duration of stent therapy, (4) the length of hospital stay, (5) the duration of intensive-care therapy, (6) the duration of parenteral nutrition and (7) the time to resumption of oral nutrition.

The success of stent therapy was defined as complete healing of the GL without changing the treatment strategy. A change in treatment strategy was recorded when the stent treatment failed and surgical resection, endoscopic vacuum therapy or an alternative intervention was necessary to achieve healing of the GL. Death occurring in the follow-up period after GL was also coded as failure of stent-therapy. As all stents were placed successfully, we did not register and report the technical success rate.

Post-operative stent-independent complications were also registered according to Clavien-Dindo Classification [31]. Stent-dependent complications were registered separately. Pulmonary complications (pneumonia, reintubation, or mechanical ventilation), renal conditions (renal insufficiency or acute renal failure), stroke, cardiovascular conditions (cardiac arrest or acute myocardial infarction), thromboembolic conditions (pulmonary embolism or deep venous thrombosis) and infectious conditions (sepsis, septic shock, or urinary tract infections) were considered medical complications.

### Statistical Analysis

All analyses were done using the PRISM 8 GraphPad software (GraphPad Software, 2365 Northside Dr., Suite 560, San Diego, CA 92108, USA). We summarized continuous variables as mean (standard deviation, SD) and median (interquartile range, IQR). Categorial variables were registered as n (%). Statistical comparisons of quantitative variables were performed using Student’s t test or Mann-Whitney test. For categorial variables, we used Pearson’s Chi^2^ test or Fisher’s exact test. All statistical tests were two-sided, and p values of less than 0.05 were considered statistically significant. For the purpose of comparing the treatment outcomes and morbidity between the stent groups (SS vs. MS), we used analogous statistical tests.

In order to address possible bias (e.g. differences between groups for age, sex), we compared demographic variables in both groups (SS and MS) (Table 1). Given that no statistical difference was detected between the groups, no further matching was necessary.

**Table 1:** Characteristics of patients treated with stent for gastric sleeve leakage

Variable	Total cohort (n = 23)	SS (n = 12)	MS (n = 11)	P value
Age, y, mean (SD)	43.9 ± 10.5	42.3 ± 12.9	45.6 ± 7.2	0.475^a^
BMI, kg/m^2^, mean (SD)	52.0 ± 10.5	50.9 ± 7.4	53.3 ± 13.4	0.591^a^
Sex, female, n (%)	12 (52.2)	7 (58.3)	5 (45.5)	0.684^b^
Hypertension, n (%)	16 (69.6)	9 (75.0)	7 (63.6)	0.667^b^
Diabetes mellitus type 2, n (%)	15 (65.2)	10 (83.3)	5 (45.5)	0.089^b^
Dylipidemia, n (%)	15 (65.2)	8 (66.7)	7 (63.6)	0.999^b^
NAFLD/NASH, n (%)	13 (56.5)	8 (66.7)	5 (45.5)	0.414^b^
ASA, n (%)				0.565^c^
II	11 (47.8)	6 (50.0)	5 (45.5)	
III	11 (47.8)	6 (50.0)	5 (45.5)	
IV	1 (4.4)	0 (0.0)	1 (9.0)	

^a^t-test for normal distributed variables; ^b^ Fisher´s exact contingency test; ^c^Chi-square test

## Results

### Participant Characteristics

**Fig. 1:**
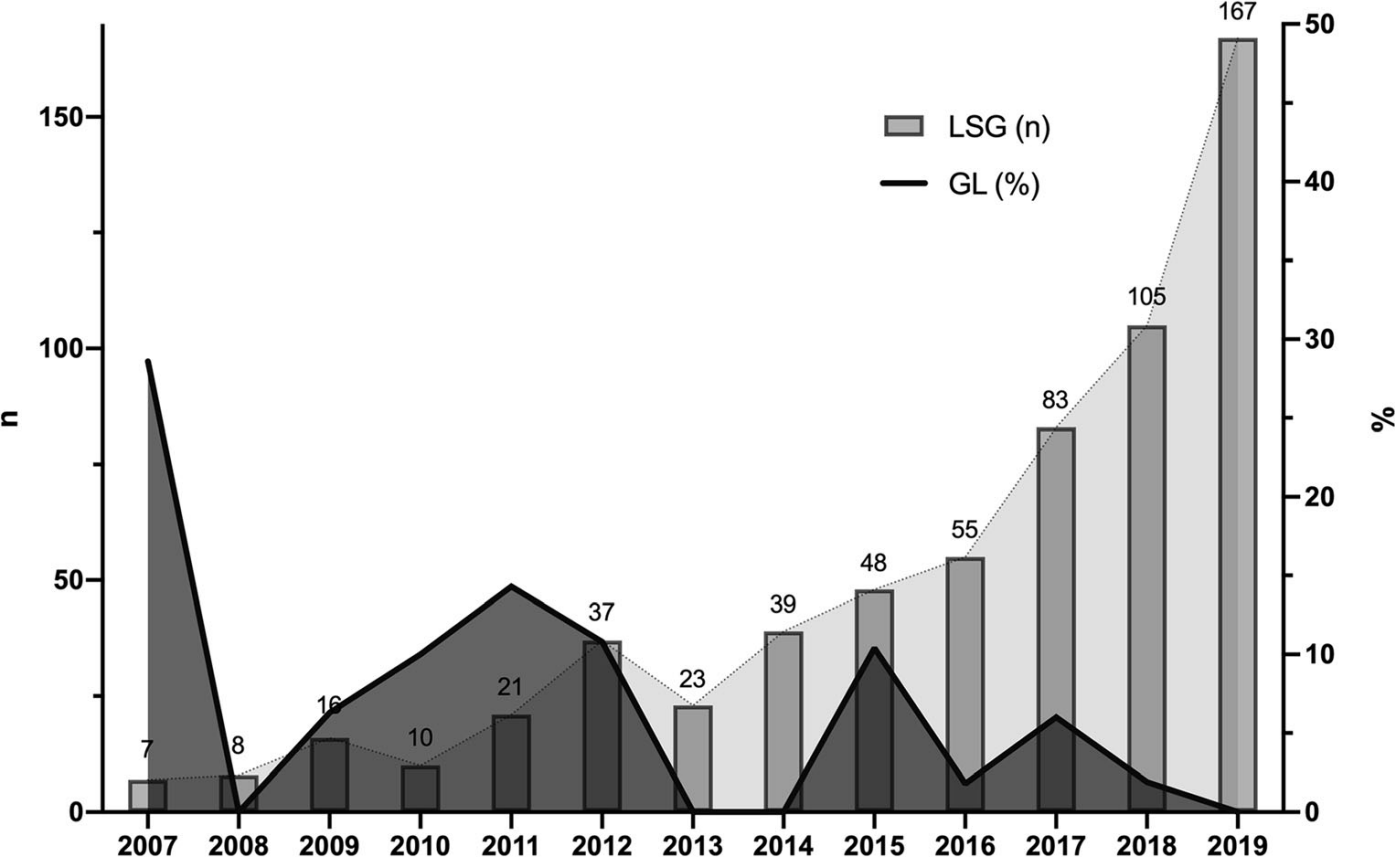
Distribution of the incidence of gastric staple line leaks (GL) in relation to the number of laparoscopic sleeve gastrectomy (LSG) procedures between 2007 and 2019

We identified 619 patients who underwent LSG (among a total of 1084 bariatric procedures) for morbid obesity in our academic surgical centre between 2007 and 2019. Over this period, 23 of these patients (3.7%) developed a post-operative GL. The distribution of the incidence of GL following LSG in relation to the number of procedures performed yearly is shown in Fig. 1. Between 2007 and 2019, the annual number of LSG procedures performed increased from 7 to 167. During the same period, the incidence of GL decreased from 28.6 to 0% reflecting our institutional learning curve. Patient characteristics are shown in Table 1. A relative stricture distal to the leak was not described in any patient of our series. The two patient cohorts analysed were (A) SS (n = 12) and (B) MS (n = 11). There were no statistically significant differences between these groups in terms of baseline patient characteristics, especially in terms of co-morbidities (e.g. diabetes mellitus, arterial hypertension, dyslipidaemia, NAFLD or metabolic syndrome). Eleven patients (47.8%) were male and 12 (52.2%) were female. Mean age was 43.9 years (SD = 10.5). Peri-operative (in relation to primary surgery) and peri-interventional (in relation to stent placement) variables are summarized in Table 2 and Table 3, showing both groups (SS vs. MS) to be comparable for these variables. Two patients treated with SS had a drain placed during primary surgery. Drain placement at the end of primary surgery was a standard of care at the beginning of our bariatric surgery program and was not related to the development of any intra-operative complications. The mean time from initial surgery (LSG) to diagnosis of GL was 9.6 days (SD = 7.3), with mean time from diagnosis of GL to stent placement of 1.2 days (SD = 1.2). Twenty out of 23 patients (87.0%) underwent one or two re-operation(s) after the diagnosis of GL. Fifteen patients had only one re-operation for wash-outs and operative drain placement. Three patients (13.0%) had two re-operations, the first of which being for wash-out and operative drain placement. The second re-operation was a subtotal gastrectomy (n = 1) to treat persistent GL, a laparoscopic gastric bypass (n = 1) to treat reflux and an open drain placement (n = 1). There was no statistically significant difference between both groups. Interventional drainage took place in 8 patients (34.8%) to treat intra-abdominal fluid collections prior to or after stent placement (as the only drainage method or as a complementary treatment to surgical drainage).

**Table 2:** Peri-interventional measures

Variable	Total cohort (n = 23)	SS (n = 12)	MS (n = 11)	P value
Interval primary surgery-diagnosis of leak, d, mean (SD)	9.6 ± 7.3	12.2 ± 9.3	6.7 ± 2.3	0.112^a^
Interval diagnosis-stent, mean (SD)	1.2 ± 1.2	1.2 ± 0.7	1.2 ± 1.6	0.839^b^
Interval diagnosis-surgical drain if placed, mean (SD)	0.3 ± 0.8	0.3 ± 0.9	0.3 ± 0.7	0.721^a^
Interval diagnosis-interventional drain if placed, d, mean (SD)	2.5 ± 3.2	3.8 ± 3.5	0.3 ± 0.6	0.150^b^
Use of drain in primary OP, n (%)	2 (8.7)	2 (16.7)	0 (0.0)	0.478^c^
Use of antibiotic prophylaxis during primary surgery, n (%)	23 (100.0)	11 (100.0)	12 (100.0)	1.000^c^
Re-operation needed after diagnosis, n (%)	20 (87.0)	11 (91.7)	9 (81.8)	0.590^c^
Use of drain in re-op, n (%)	21 (100.0)	12 (100.0)	9 (100.0)	1.000^c^
Use of interventional drain, n (%)	8 (34.8)	5 (41.7)	3 (27.3)	0.667^c^
Use of antibiotics after diagnosis of leak, n (%)	23 (100.0)	12 (100.0)	11 (100.0)	1.000^d^
Use of antibiotics after stent placement, n (%)	23 (100.0)	12 (100.0)	11 (100.0)	1.000^d^

^a^Mann Whitney test; ^b^ Student t test; ^c^ Fisher’s exact test; ^d^ Chi square test

**Table 3:** Primary outcomes and key secondary outcomes

Variable	Total cohort (n = 23)	SS (n = 12)	MS (n = 11)	P value
Stent treatment success, n (%)	16 (69.6)	6 (50.0)	10 (90.9)	**0.006**^**a**^
Stent complications, n (%)				
Migration	7 (30.4)	6 (50.0)	2 (18.2)	0.193^a^
Stenosis/stricture	3 (13.0)	2 (16.7)	1 (9.1)	0.999^a^
Intolerance	3 (13.0)	2 (16.7)	1 (9.1)	0.999^a^
Extraction due to intolerance	3 (13.0)	2 (16.7)	1 (9.1)	0.999^a^
Perforation/bleeding	0 (0)	0 (0)	0 (0)	1.000^a^
Stent-independent complications, n (%)				
Clavien-Dindo Grad 1	0 (0.0)	0 (0.0)	0 (0.0)	1.000^a^
Clavien-Dindo Grad 2	0 (0.0)	0 (0.0)	0 (0.0)	1.000^a^
Clavien-Dindo Grad 3A	1 (4.3)	0 (0.0)	1 (9.1)	0.478^a^
Clavien-Dindo Grad 3B	15 (65.2)	9 (75.0)	6 (54.5)	0.400^a^
Clavien-Dindo Grad 4A	4 (17.4)	2 (16.7)	2 (18.2)	0.999^a^
Clavien-Dindo Grad 4B	0 (0.0)	0 (0.0)	0 (0.0)	1.000^a^
Clavien-Dindo Grad 5	2 (8.7)	1 (8.3)	1 (9.1)	0.999^a^
Mortality, n (%)	2 (8.7)	1 (8.3)	1 (9.1)	0.999^a^
Nutrition after stent placement, n (%)				
Suppl. enteral feeding (tube)	13 (56.5)	10 (83.3)	3 (27.3)	**0.012**^**a**^
Suppl. parenteral feeding (iv)	19 (82.6)	11 (91.6)	8 (72.7)	0.317^a^
Duration of parenteral nutrition if needed, mean (SD), d	20.0 ± 29.1	32.4 ± 36.3	9.5 ± 5.4	**0.009**^**b**^
Duration of enteral feeding if needed, mean (SD), d	35.8 ± 32.8	44.5 ± 32.6	6.7 ± 5.7	0.078^c^
Start of per os nutrition (after stent placement), mean (SD), d	12.2 ± 25.4	23.1 ± 33.1	1.4 ± 1.1	**0.003**^**b**^
Duration of hospital stay, mean (SD), d	59.9 ± 70.5	95.9 ± 83.0	20.6 ± 10.6	**< 0.0001**^**b**^
Duration ICU stay, mean (SD), d	10.2 ± 32.3	14.4 ± 42.4	5.8 ± 16.2	0.598^b^
Duration of stent therapy, mean (SD), d	65.4 ± 54.7	83.3 ± 70.1	44.0 ± 5.8	0.290^b^
Total number of stents, mean (SD)	1.6 ± 0.8	2.1 ± 0.9	1.0 ± 0.0	**0.0003**^**b**^
Total number of endoscopies, mean (SD)	4.6 ± 3.5	6.8 ± 3.7	2.3 ± 0.5	**< 0.0001**^**b**^

^a^Fisher’s exact test; ^b^Mann-Whitney test; ^c^ Student t test; **bold:** statistical significant

### Success Rate of Stent Therapy

Endoscopic stent therapy was a successful in 16 patients (69.6%) out of 23 in the total cohort. MS therapy was associated with a significantly higher rate of success of 90.9% (compared to 50.0% in the SS group; p = 0.006). Stent failure was due to (i) patient death (2 patients died, one in the MS and one in the SS cohort) and (ii) change in treatment strategy due to persistent leak (in 3 patients in the SS cohort, the treatment was changed from stent to conservative/E-VAC and in 2 patients in the SS cohort, the treatment was changed to MS).

### Morbidity and Mortality

Two deaths (8.7%) were observed in our series: one patient in the SS group who died after multiorgan failure due to gallbladder empyema and pneumonia, and one patient in the MS group who died after severe heart failure. This patient had multiple co-morbidities with a Charlson Comorbidity Index of 9. This mortality was coded as stent failure, although the death was not directly related to a failure of stent therapy. Autopsy confirmed that the GL was adequately covered by the MS and there was no perigastric inflammation.

We differentiated between complications related to stent placement and other stent-independent complications. Stent-independent complications were classified according to the Clavien-Dindo classification [31] and appear in Table 3.

Seven patients (30.4%) in our cohort were diagnosed with stent migration that required endoscopic correction to reposition the stent. In three patients (13.0%), the stent was not tolerated and had to be removed; the GL healed in these patients without need of change in treatment strategy. In all three patients, the fistula had either disappeared following stent treatment or was reduced in size and no longer required further treatment. In all these cases, it was decided to simply observe the patients with regular endoscopic examinations. Three patients (13.0%) from the entire cohort developed a stricture/stenosis. The strictures were treated by endoscopic dilatation and one patient required re-stenting. There were no cases of bleeding or perforation in our cohort. There were no significant differences between the SS and MS groups in terms of stent-related complications.

### Nutrition

All patients treated with MS (n = 11, 100.0%) were able to resume oral nutrition within 3 days of stent placement, while only 3 out of 12 patients (25.0%) treated with SS were able to do so. The time between stent placement and resumption of oral nutrition was 12.2 days in the whole cohort and only 1.4 days in the MS group. MS patients had a significantly earlier return to oral nutrition in comparison to SS patients (1.4 vs 23.1 days; p = 0.003). The need for, and duration of, supplemental enteral or parenteral feeding is shown in Table 3. Enteral tube feeding had to be used significantly more often in the SS group (83.3 vs. 27.3%, p = 0.012). In contrast, MS patients required parenteral nutritional support for a significant shorter period (9.5 vs 32.4 days, p = 0.009).

### Number of Endoscopies and Stents

The number of endoscopies and stents needed to achieve healing in both cohorts are shown in Table 3. A mean of 1.6 ± 0.8 stents were needed to achieve healing of the GL in the present series, with an average of 4.6 ± 3.5 endoscopies performed in total. MS patients needed significantly less stents (actual physical number of stents) in comparison to SS patients to achieve treatment success (1.0 vs. 2.1 stents; p = 0.0003). As with the number of stents, significantly less endoscopic procedures were performed in MS patients in comparison to SS patients (2.3 vs. 6.8; p < 0.0001). The total duration of stent therapy was 65.4 ± 54.7 days for the whole cohort. Although this treatment duration was shorter in the MS group, the difference did not reach significance (44.0 vs. 83.3 days, p = 0.290).

### Hospital Stay and Duration of ICU Stay

Hospital stay and duration of ICU therapy are shown for both cohorts in Table 3. For our cohort as a whole, the mean hospital stay was 59.9 ± 70.5 days. The group of patients treated with MS showed a significant shorter hospital stay than the group treated with SS (respectively 20.6 ± 10.6 vs. 95.9 ± 83.0 days; p < 0.0001). The duration of ICU stay was 10.2 ± 32.3 for the whole cohort and comparable between both groups (respectively 5.8 ± 16.2 vs. 14.4 ± 42.4; p = 0.598).

## Discussion

Our data support the current evidence that stent therapy in general is an effective and safe treatment GL following LSG, not only allowing early resumption of oral feeding, but also keeping the period of hospitalization to a minimum. Furthermore, MS may provide additional advantages when compared to SS, namely increased rates of treatment success coupled with a reduction in the numbers of endoscopies and stents required to achieve healing of the GL. Chronic leaks were not observed in our study and, therefore, the conclusions proposed in our series cannot be applied to this complication. The lack of chronic fistulas in our series could even be the result of a successful stent treatment strategy.

Endoscopic stent implantation successfully treated GL in 69.6% of our patients. This result is consistent with published studies (success ranging from 65 to 100%) [9–22, 32] and with systematic reviews (up to 87.8%) [33]. However, in cases where stent therapy was performed with a MS, the success rate was significantly higher (90.9 vs. 50.0% respectively, p = 0.006), which is also in line with the available literature [10–13, 15–20, 34–40]. This suggests that the success of stent therapy for GL is at least partially dependent on sufficient bridging of the area between the esophagus and the duodenum. In combination with the sealing effect of the stent, this may also reflect successful dilatation of a possible (relative) stenosis distal to the fistula (GL is frequently the consequence of such a stenosis) (i.e. stent may restore the balance of pressure between the proximal and distal parts of the stomach) afforded by the stent [25, 33, 41]. In turn, this may facilitate healing in a more uniform manner due to equilibration of intraluminal pressure and also underpin the benefits of MS over SS, given that the latter only affords partial coverage of the gastric sleeve. To our knowledge, however, there are no studies to date that have shown that MS has been able to restore pressure equilibrium in the gastric compartment per se.

Stent migration is the most frequently reported complication after stent placement, with an incidence cited between 17 and 29% in large series and reviews [9, 11–13, 16, 18–20, 25, 34–37, 39, 42]), and often necessitates stent re-positioning. Megastents were developed with large stent flanges to prevent migration and several authors have observed significantly less migration with this type of stent (0–21.6%) [11, 15, 16, 36] when compared to SS (6–60%) [9, 10, 18, 34, 37, 39]. Several advantages of MS were also evident in our series with a lower incidence of migration, although the result did not reach statistical significance (18.2% for MS vs. 50.0% for SS, p = 0.193). Stent intolerance occurred in 9.1% of MS and 16.7% of SS treated patients, requiring removal of the stents in all cases. Therefore, we could not confirm the major reported criticisms of MS, i.e. abdominal pain in 15–97% of patients and removal in 11-15% [15, 16, 36, 37, 39]). Bleeding and perforation are also recognised complications of stenting, with incidences in the literature [25, 27, 32, 43] ranging respectively from 0 to 11.8% and from 0 to 8.4%. These complications were not encountered in our patients. We observed stricture development in 16.7% of cases after SS therapy and 9.1% after MS, comparable to the data reported in the literature [9, 11, 13, 15, 16, 36, 37, 39]. We could not retrospectively assess stent type-dependent difficulties in extraction, given that this information was not routinely recorded.

Endoluminal vacuum therapy has recently been proposed as alternative treatment option for GL [44, 45], primarily based on its efficacy in the management of anastomotic leaks following esophageal surgery. However, the evidence for the use of E-Vac in the specific management of sleeve GL remains the majority of the data supporting E-Vac therapy derives from studies in heterogenous patient cohorts, for a range of conditions [44–53]. In our hands, the results of EVac therapy for GL have been disappointing. Therefore, this procedure is only used as a bridging therapy when patient is presenting at night in the emergency setting, allowing us to place the stent on an elective list the day after in a well-controlled setting. Future studies may be well advised to specifically assess the success rates and other outcomes of each of these treatment modalities.

Stent therapy allowed early resumption of oral nutrition (after 12.2 days (1.4 days for MS alone)) in our series, which is essential to promote healing. Early resumption of oral nutrition is superior to parental nutrition in order to achieve this goal in the most effective and least expensive fashion [54]. Parenteral nutrition was required for an average of 20.0 days (only 9.5 days when only MS are considered), consistent with the published literature [4, 11, 16–18, 25, 35]. It is worth bearing in mind that timely resumption of oral intake [11, 16–18, 55] is not possible with E-Vac treatment, given that oral feeding is only possible after completion of the therapy, i.e. on average 23–50 days [44, 45, 52, 56].

Other important factors when determining the most effective treatment of GL following LSG are the number of endoscopies and stents required before healing is complete. Not only do these factors impact heavily upon patient well-being, but they are also associated with significant economic costs. In line with the literature [9, 11, 13, 15, 16, 18, 20, 26, 32, 34, 38], the average number of endoscopies and stents in our series were 4.6 and 1.6 respectively. There was a significant difference when comparing the MS to SS group (2.3 ± 0.5 vs. 6.8 ± 3.7 for the number of endoscopies, p = 0.001 and 1.0 ± 0.0 vs. 2.1 ± 0.9 for the number of stents, p = 0.0003), suggesting that MS may be preferable to SS, both in terms of efficacy and cost. In contrast, the number of endoscopies required for E-Vac therapy ranges from 7 to 18 [44, 45, 52].

It is essential to differentiate between the length of hospital stay and the time taken to successful healing of the GL, which we defined as the length of active stent therapy. In our series, patients were hospitalized for an average of 59.9 days, while the average length of stent therapy was 65.4 days. These durations are consistent with those previously published (i.e. with hospitalizations between 20 and 90 days and stent treatment durations between 15 and 75 days) [9, 12, 16, 17, 20, 34–38]. Patients treated with MS had even shorter treatment durations (44.0 days for MS vs. 83.3 days for SS in our series) (15–50 days in the literature). There was however no statistically significant difference between MS and SS groups.

The overall incidence of GL after LSG (3.7%) observed in our series was broadly comparable to that reported in recent studies [9–22]. The incidence decreased dramatically from 28.6% in 2007 to no cases in 2019. It is conceivable that this reduction was partially due to the increased experience with the procedure acquired in our centre. Although there was a difference in the interval between primary surgery and diagnosis of GL (12.2 ± 9.3 days vs. 6.7 ± 2.3 days for SS and MS, respectively), this was not statistically significant. The difference may have been an incidental finding or reflected an experience-dependent improvement in the early diagnosis of GL in our centre. With growing experience, we were more attentive to signs of GL. A prolonged delay between primary surgery and GL diagnosis may, in theory, lead to an aggravation of a localized infection into sepsis. However, by analysing the stent-independent complications, we observed that both groups were comparable. Therefore, there was no evidence that this potential difference influenced outcomes. While definitive treatment of GL centres on endoscopic stent placement, abdominal collections must be drained (radiologically, surgically or endoscopically) before or after stenting [46, 57–60]. The delay of 1.2 days between the diagnosis and stenting of GL may have been due to the fact that patients initially underwent wash-out and drainage placement or interventional drainage. When surgery was scheduled, no other intraoperative manoeuvres other than drain placement were undertaken (e.g. leak suture). In recent years, we have moved from surgical to interventional CT-guided drainage in order to enable earlier stenting and to reduce the delay between GL diagnosis and treatment. In addition, surgical drainage runs the risk of disseminating infection, allowing a local intra-abdominal infection focus to develop into peritonitis and/or sepsis. In fact, this may have contributed to the sepsis and the lethal multi-organ failure seen in one of our patients. This patient was recorded as a treatment failure, although his pre-existing co-morbidities (congestive heart failure) and GL likely were more contributory to his death than stent implantation. The second fatality occurred one year after the diagnosis and therapy of GL. The patient was readmitted with septic shock due to gallbladder empyema and pneumonia; despite maximum intensive care treatment, the patient developed multiorgan failure and died. In line with published literature [43], our data confirmed GL as a potentially life-threatening complication of LSG (mortality = 8.7%).

## Limitations

A key limitation of our study is its retrospective design, which introduces the potential for selection bias. However, there were no statistically significant differences between the MS versus SS groups in terms of baseline patient characteristics and our cohort was comparable to those reported in the literature. Another inevitable consequence of retrospective observational research is the potential risk of missing data, as the availability of baseline and outcome data is largely dependent on the completeness of medical records. We did not use methods to correct non-response bias (e.g. a multiple imputation method) given the significant sample size and that the data was well recorded. Furthermore, we were unable to perform a cost analysis as costs were not comprehensively documented.

With regard to outcome data, no patient was lost to follow-up during the entire treatment period until recovery from GI or death. Thus, the risk of underestimating morbidity or mortality was low. A detailed examination of the long-term complications after stent insertion or the type of operation performed, although clinically highly relevant, was not possible due to the retrospective nature of the study. Therefore, we cannot draw any conclusions on whether the type of stent used may influence the incidence of long-term complications.

Finally, the monocentric setting of the present study is reflected in the relatively small number of patients with GL that were identified. However, the advantage of the monocentric setting was that the cohort was homogenous in terms of baseline characteristics and all patients were followed-up, assessed and operated upon by the same surgeons and following the same standards of care. This means that the risk of chronological bias was kept to a minimum.

## Conclusion

Stent therapy is an effective and safe treatment of GL following LSG. MS insertion may offer several advantages over SS, perhaps related to the degree of coverage (complete bridging) obtained between the esophagus and the duodenum. Specifically, MS insertion allows early resumption of oral nutrition and keeps the period of hospitalization to a minimum. Furthermore, compared to SS, it led to a reduction in the numbers of endoscopies and stents needed to achieve healing. Our data highlight that successful stent-therapy depends on a multimodal treatment algorithm based on endoscopy, stent selection and implantation, drainage of fluid collections (surgical or CT-guided), nutritional- and intensive care support. In the majority of GL patients, this treatment concept was successful. Of course, the algorithm needs to be adapted depending on the patient’s clinical presentation, co-morbidities and overall state of health. Future studies are required comparing our multimodal treatment concept with other treatment regiments. At least at present, the current evidence base favours endoscopic stenting for the management of GL, but further studies should specifically examine whether novel interventions, including E-Vac therapy, lead to any cost-effective therapeutic advantages.

## Abbreviations

ASA: American Society of Anesthesiologists – Physical Status Score
BMI: body mass index
CAADIP: Chirurgische Arbeitsgemeinschaft Adipositastherapie und Metabolische Chirurgie (Surgical Working Group for Obesity Therapy amd Metabolic Surgery)
DGAV: Deutsche Gesellschaft für Allgemein- und Viszeralchirurgie
DPS: double pigtail stent
E-Vac: endoscopic vacuum therapy
GL: gastric leak (or gastricstaple line leak or gastric staple line insufficiency)
IFSO: International Federation for the Surgery of Obesity and Metabolic Disorders
LGB: laparoscopic Roux-en-Y gastric bypass
LSG: laparoscopic sleeve gastrectomy
MIS: minimally invasive surgery
MS: Mega Stent
NAFLD: non-alcoholic fatty liver disease
NASH: non-alcoholic steato-hepatitis
SD: standard deviation
SS: small stent
